# Characterizing mixed strongyle infections in foals and broodmares using cytochrome *c* oxidase subunit I deep amplicon sequencing

**DOI:** 10.1186/s13071-025-07192-1

**Published:** 2026-01-03

**Authors:** Luise Grace Klass, Jürgen Krücken, Susan Mbedi, Sarah Sparmann, Thore Schenk, Sandro Andreotti, Georg von Samson-Himmelstjerna

**Affiliations:** 1 Institute for Parasitology and Tropical Veterinary Medicine, Freie Universität Berlin, Berlin, Germany; 2 Veterinary Centre for Resistance Research, Freie Universität Berlin, Berlin, Germany; 3 Present Address: Institute of Veterinary Anatomy, Freie Universität Berlin, Berlin, Germany; 4 Berlin Center for Genomics in Biodiversity Research (BeGenDiv), Berlin, Germany; 5 Bioinformatics Solutions Center, Institute of Computer Science, Freie Universität Berlin, Berlin, Germany; 6 Leibniz Institute of Freshwater Ecology and Inland Fisheries (IGB), Berlin, Germany; 7 Leibniz Institute for Zoo and Wildlife Research, Berlin, Germany

**Keywords:** Nemabiome, Strongylidae, Strongylid, Equine helminths, Cyathostominae, Cytochrome *c* oxidase subunit I, High-throughput sequencing, Next-generation sequencing

## Abstract

**Background:**

Mixed strongyle infections represent the most prevalent equine parasitosis and can result in life-threatening disease, especially in young horses. Species involvement and pathogenesis of this parasitosis are poorly understood, and data on foals and broodmares are notably lacking.

**Methods:**

In a longitudinal study undertaken in 2022 in Germany, individual faecal samples (*n* = 497) and metadata were collected for naturally infected foals and broodmares (*n* = 48) kept under conventional husbandry conditions. Nematode infections were detected coproscopically via the Mini-FLOTAC method. In a subset of strongyle egg-positive samples (*n* = 46), species were identified using cytochrome *c* oxidase subunit I deep amplicon sequencing. Species prevalence, richness, and alpha and beta diversity were compared between foals and mares.

**Results:**

Overall, 22.2% of the foal samples and 10.2% of the mare samples were strongyle egg positive (eggs per gram > 5). *Parascaris* spp. were only detected in foals (15.1%). *Strongyloides westeri* was detected in one foal sample. Strongyle egg detection increased in likelihood with each additional sample timepoint (OR = 1.42, *P* < 0.001) and with ascarid egg detection (OR = 6.49, *P* < 0.001), while last anthelmintic treatment with pyrantel decreased the odds of detecting eggs (OR = 0.12, *P* = 0.002). Deep amplicon sequencing detected 16 species of small strongyles but no large strongyle species. *Cylicostephanus goldi, Cylicostephanus minutus* operational taxonomic unit II and *Cylicocyclus ashworthi* were significantly more prevalent in mares (*P* < 0.05), while *Cylicostephanus calicatus* operational taxonomic unit II was more prevalent in foals (*P* < 0.01). Mares showed a significantly higher amplicon sequence-variant-based richness (Chao 1 index, *P* < 0.001) and diversity (inverse Simpson index, *P* < 0.01) than foals. Group (foals vs. mares) explained some of the variance in beta diversity, according to permutational multivariate ANOVA. Co-infection with *Parascaris* spp. did not affect strongyle community composition in the foals. Bray–Curtis and Jaccard distance (dissimilarity) plots showed separate clusters for mares and foals, with some overlap and a moderate model fit.

**Conclusions:**

Cytochrome oxidase-based characterization of mixed strongyle infections revealed strongyle community differences between broodmares and foals. Possible age associations were identified for four species of small strongyles, including two cryptic species. Low overall strongyle prevalence and egg-shedding intensity, non-random sampling and differences in anthelmintic treatment schemes limited the statistical power of this study.

**Graphical Abstract:**

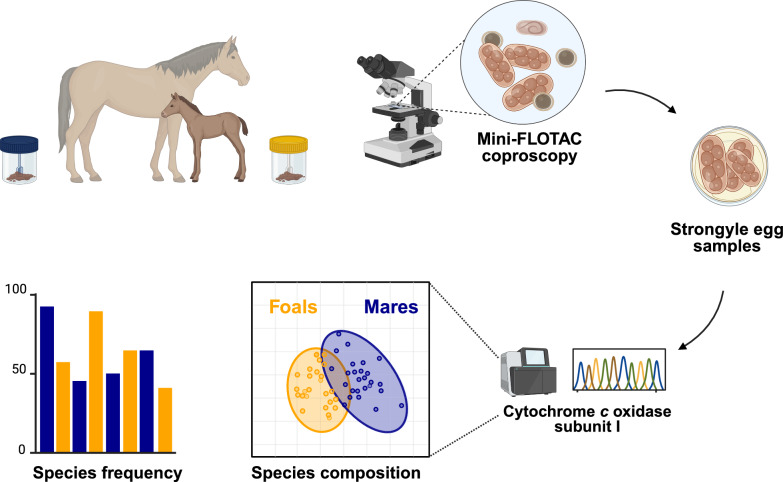

**Supplementary Information:**

The online version contains supplementary material available at 10.1186/s13071-025-07192-1.

## Background

Mixed strongyle infections are the most common parasitoses of equines worldwide [1–5]. Among these parasites, small strongyles are the most prevalent, and typically occur as multi-species infections, often with 10–20 species per host. While most small-strongyle infections remain subclinical, larval cyathostominosis can be life-threatening, and particularly affects young horses [6–9]. In fact, among fatal cases of parasitosis caused by strongyles, cyathostominosis has taken over as the leading cause of death (by *Strongylus vulgaris*) of equines in some European countries [4]. Yet, which precise species are involved and their pathogenicity are poorly understood, and data for foals are currently lacking.

To date, 64 strongyle species have been recognized, based on their morphological identification [10], of which 35 have been detected in domestic horses over the past 50 years [3]. Small-strongyle infections are dominated by a core group of 10–12 species, which are the most prevalent and typically most abundant species of strongyles [3, 11]. Species richness varies between infected individuals and has been linked to age group and geographical region [3]. In a meta-analysis of data from Western Europe [3], cyathostomin communities comprised an average of 18 species and were dominated by the following: *Cylicocyclus (Cyc.) nassatus*, *Coronocyclus (Cor.) coronatus*, *Cylicostephanus* (*Cys.) longibursatus*, *Cyathostomum* (*Cya.) catinatum*, *Cylicostephanus* (*Cys.) minutus*, *Cylicostephanus* (*Cys.) goldi*, *Cylicocyclus (Cyc.) insigne, Coronocyclus (Cor.) labiatus*, *Coronocyclus (Cor.) labratus*, *Cylicocyclus (Cyc.) leptostomum* and *Poteriostomum imparidentatum.* While generally thought to share similar characteristics, differences in these species’ biology have been described. This includes differences in prepatent periods between species [11] and shortened post-treatment egg-reappearance periods [12–15], with unknown consequences for the host. Moreover, anthelmintic resistance is widespread among small strongyles, especially against benzimidazoles [3, 16, 17], but also against tetrahydropyrimidines [18, 19] and macrocyclic lactones [20–22]. Some studies [5, 12, 18–20, 23] have associated certain species with anthelmintic resistance against certain classes of drugs, which highlights the importance of characterizing mixed strongyle infections. In horses, differences in species composition have been described between both individuals and age groups, and also between farms, and regions [5, 24–27]. Moreover, the influence of the sex of the host, weather and seasonality on species composition have been studied and discussed [5, 26–31]. However, diagnostic challenges limit the overall availability of data, and our understanding of this complex nematode subfamily, the Cyathostominae, is still incomplete [1, 32].

Gastrointestinal parasite infections in horses are routinely diagnosed through faecal egg counts (FEC) [33]. The Mini-FLOTAC method employs a low multiplication factor for the counting of eggs compared with, for example, the McMaster method, and has been found to be more precise and sensitive than the latter [34, 35]. It is frequently used for the detection of all common equine nematodes encountered in continental Europe, including migratory and non-migratory Strongylidae (for which the eggs are detected), *Strongyloides westeri, Parascaris* spp.*, Oxyuris equi* and, with less sensitivity, Anoplocephalidae [36–39]. Egg flotation tests allow for a wide range of parasite species to be differentiated. However, strongyle eggs share the same morphology across all species of the Strongyloidea and Trichostrongyloidea, necessitating additional diagnostic measures [1]. Deep amplicon sequencing has been proven to be a useful and cost-effective method for the differentiation of strongyle species [1, 5, 32, 40–43], with three target gene sequences currently in use. Internal transcribed spacer 2 (ITS-2) has been used for nemabiome analysis in equines [5, 18, 20, 32, 40, 41, 44–47], but although this is a well-established target amplicon, it cannot reliably differentiate two very common and closely related core species of small strongyles, *Cor. coronatus* and *Cys. calicatus* [1]. Two cytochrome *c* oxidase subunit I (COI) target amplicons have also been used to differentiate equine strongyles, a short 450-base pair (bp) target [40] and, more recently, a long 650-bp target [43]. While the 450-bp COI target amplicon showed an inferior performance with ITS-2 when applied to a mock community [40], the longer 650-bp COI target amplicon can differentiate all core species of small strongyles described for continental Europe [43]. Furthermore, approaches employing both ITS-2 [32] and long COI [42, 43, 48] have been used to detect large strongyles. To date, our COI reference database includes sequences for 30 strongyle species obtained from individually morphologically identified specimens, for which paired COI and ITS-2 data are available. These include all cryptic species described for *Cys. calicatus* operational taxonomic units (OTUs) I–III and *Cys. minutus* OTUs I–III [13, 42, 43, 49]. Metabarcoding data for foals and broodmares are lacking and, moreover, there are only few data available on the occurrence of species of small strongyles in horses in Germany [43].

The aim of this study was to investigate natural mixed strongyle infections in foals and broodmares in Germany managed under conventional husbandry conditions. Specifically, the goals were to identify factors influencing strongyle egg detection and strongyle species composition, as well as strongyle community structure.

## Methods

### Study population

This longitudinal study was conducted in Germany during the 2022 grazing season (May–November) on three commercially operated breeding farms of warmbloods (*Equus ferus caballus*) located in the states of Brandenburg, Hesse and Bavaria. In total, 497 individual faecal samples were collected from 48 horses (*n* = 24 mare-foal pairs). The mares were 4–23 years old at the time of sampling (mean, 11.3 years; median, 10 years). The foals were 25–99 days old (mean, 52 days; median, 47 days) at the first sampling timepoint and 121–239 days old (mean, 177 days; median, 176 days) at the last sampling timepoint. Farms were selected based on their expected number of warmblood breed foals (*n* = 5–20), due dates (March–May), husbandry practices (individual stalls and daily grazing), absence of planned antibiotic treatments and absence of reoccurring health issues among the horses, e.g. a history of *Rhodococcus equi* on the farm. For each of the farms, all healthy foals and their dams were included in the study. Daily access to the grazing pasture started 2–6 weeks postpartum and lasted for the duration of the study (May–November).

All medical treatments, starting from birth, as well as general husbandry data, were recorded. Anthelmintic treatment followed normal farm practices. For foals, this consisted of a single fivefold dose of fenbendazole (50 mg/kg body weight) around 7–14 days postpartum, followed by one single-fold dose of fenbendazole (7.5 mg/kg body weight) or pyrantel embonate (19 mg/kg body weight) at 8- to 10-week intervals, starting at 6–8 weeks of age. For mares, a single dose of ivermectin (0.2 mg/kg body weight) was given 7 days prior to the due date or on the day of parturition, followed by pyrantel embonate, ivermectin or moxidectin (0.4 mg/kg body weight) at 3-month intervals. To determine the anthelmintic dosage for the adult horses, the body weight, which was estimated by the stable management, was increased by adding 50 kg. The mares were between 162 and 175 cm wither height and estimated to weigh between 550 and 700 kg after parturition. The foals were expected to mature to similar sizes and weights, and a birth weight of 50 kg was assumed for anthelmintic dosage purposes.

### Collection of faecal samples

A total of 497 individual faecal samples were collected for the 11 timepoints, and comprised 260 samples from foals and 237 samples from mares. All faecal samples were obtained from naturally passed faeces and collected from the centre of a fresh pile, to prevent environmental contamination. Prior to weaning, each mare-foal pair was kept in an individual stall with straw bedding for periods of the day, and at least during sample collection, and were otherwise grazing on pasture. After weaning, two to three foals were grouped in one stall. Before weaning, large faecal samples (> 100 g) for parasitological testing were obtained in the mornings and kept at 4–10 °C until further processing. Differentiation between mare and foal faeces was easily achieved by visual assessment of size, colour and texture. For weaned foals housed in pairs or groups, defecation was monitored closely to ensure faeces of the correct individual were sampled.

### Faecal egg counts

Faecal egg counts were performed for all samples (*n* = 497; stored at 4–10 °C) within 3 days of sampling. The Mini-FLOTAC method was applied to count nematode eggs [37, 50]. In brief, 5 g of faeces was mixed with 45 ml saturated NaCl solution (specific gravity 1.20) and strained through a sieve. After mixing, the filtrate was aspirated with a pipette and both counting chambers of the Mini-FLOTAC device were filled completely. After 10 min of flotation, the upper part of the device was rotated by 90° relative to the base and all of the eggs in both chambers of the Mini-FLOTAC device were counted per egg type and the number multiplied by 5 to calculate eggs per gram (EPG).

### Egg and DNA isolation

Strongyle-positive samples were conserved in 2% Lugol’s iodine solution at a 2:1 volume:weight ratio [Lugol’s:faecal sample (millilitres:grams)], as previously described [51]. Conserved samples were stored for up to 12 weeks at room temperature before egg isolation. Strongyle eggs were successfully isolated from 64 of 81 strongyle-positive samples (strongyle EPG > 5) by washing the faeces with water over a series of sieves (500 > 180 > 60 > 25 µm). The particles remaining on the final sieve were collected into a new tube and transferred to a 25-µm sieve with a smaller diameter (7 cm) to remove as much small particulate matter as possible. The remaining residue was collected into a new 50-ml tube and purified through two rounds of flotation in saturated NaCl solution, supernatant collection and subsequent washing steps. Finally, 10 µl of the final sample was used for the microscopic count and estimation of the total number of isolated eggs. 

DNA was extracted from the samples containing > 500 eggs by using the NucleoSpin Soil Kit (no. 740780; Macherey–Nagel, Germany). To increase the retention of DNA, a modified version of the manufacturer’s protocol was applied, resulting in two extractions per sample. In brief, following mechanical beating of the eggs, the entire supernatant was divided onto two spin columns, instead of using a portion of the supernatant on one column (which is the standard protocol). The final eluates of both spin columns were collected into a single tube. All other steps followed the standard protocol. During the pre-elution incubation (3 min), the columns were maintained in a heat block (70 °C).

### Library preparation and deep amplicon sequencing

Prior to library preparation, the presence of amplifiable nematode DNA was verified by using a polymerase chain reaction (PCR) targeting the 28S ribosomal RNA gene [51]. To produce amplicon libraries that could be sequenced on an Illumina platform, a two-step PCR approach was used based on the 16S Illumina Demonstrated Library Prep Guide. This approach has been described previously [52]. Briefly, the target primers (forward primer TCG TCG GCA GCG TCA GAT GTG TAT AAG AGA CAG GAA AGT TCT AAT CAT AAR GAT ATT GG; reverse primer GTC TCG TGG GCT CGG AGA TGT GTA TAA GAG ACA GAC CTC AGG ATG ACC AAA AAA YCA A) contain Illumina adapter overhang nucleotide sequences (Technologies, Skokie, IL.) and amplify a 650-bp region of the COI in parasitic nematodes [53]. Representative reads matched to the mitochondrial COI of *Cylicostephanus minutus* OTU I (GenBank MH460767.1) approximately at positions 1–275 (273/275, 99% identity) and 379–654 (276/276, 100% identity). PCR 1 had a final concentration of 10 µM each of the forward and reverse primer, 2 µl of template DNA and 1 × (= 2.5 mM MgCl_2_) Kapa HiFi HotStart ReadyMix (Roche Molecular Systems, Peasanton, CA). The thermal cycling consisted of initial denaturation at 95 °C (3 min), followed by 34 cycles of denaturation at 98 °C (20 s), annealing at 60 °C (15 s) and extension at 72 °C (40 s), and a final elongation step at 72 °C (2 min). Magnetic beads were used to purify PCR 1 products (CleanNGS; GC biotech, the Netherlands), following the manufacturer’s instructions, using a 10:8 sample-to-bead ratio. During this process, target PCR products are reversibly attached to magnetic beads and thereby separated from excess nucleotides and non-target fragments during multiple washing steps [54]. Clean PCR products were eluted in 10 µl of water. In the second PCR step, unique dual indices (8 bp), provided by the Berlin Centre for Genomics in Biodiversity Research, were attached to each sample and the Illumina adapter was completed to allow for sequencing (Supplemental Table 7). The reaction contained purified PCR 1 product (10 µl), a unique combination of p7 and p5 indexes at a final concentration of 10 µM each, and 1 × Kapa HiFi HotStart ReadyMix (Roche Molecular Systems). For PCR 2, the thermal cycling consisted of initial denaturation at 98 °C (45 s), followed by seven cycles of denaturation at 98 °C (15 s), annealing at 60 °C (30 s), extension at 72 °C (30 s) and a final elongation step at 72 °C (1 min). To improve the yield, PCR 2 was run in duplicate. Both duplicates of PCR2 from each sample were combined into a single tube during the subsequent magnetic bead clean-up, which was performed as described above and repeated once more. Prior to sequencing, the amplicon content was quantified using a Qubit 4 Fluorometer (Thermo Fisher Scientific, Darmstadt, Germany) and the Qubit dsDNA HS Assay Kit (Thermo Fisher Scientific). Prior to sequencing, an additional magnetic bead-cleaning step was performed by using a Biomek i7 Automated Washstation (Beckman Coulter, Brea, CA). All of the samples were sequenced at the Berlin Centre for Genomics in Biodiversity Research by using Illumina MiSeq and the Version 600-Cycles Sequencing Kit (Illumina, 2 × 300 cycles). The samples were pooled according to the number of reads they produced in an initial spike-in (~ 1 million reads), and subsequently run until at least 10,000 reads per sample were obtained.

### Taxonomic assignment using DADA2 and vsearch syntax

Preprocessing of sequencing results was conducted as described previously [42, 55]. The key bioinformatic scripts are available at 10.6084/m9.figshare.29108408. Sequencing data are available from BioProject PRJNA1284338. In brief, all of the sequences resulting from the multiple runs were merged for 62 individual samples. Cutadapt was used to trim the primers and for the subsequent removal of untrimmed reads [56]. Quality checks and filters were applied, following the dada2 pipeline (R dada2 package, version 1.26.0) [57]. Reads were truncated to 260 bp and 220 bp for forward and reverse reads, respectively. The justConcatenate mode of the DADA2 mergePairs function was used, as the COI-amplicon length significantly exceeded the sum of the paired-end reads. Instead of calculating overlaps for denoised reads, this function concatenates forward and reverse reads, separated by 10 ‘*n*’. Only amplicon sequencing variants (ASVs) that matched within the family Strongylidae were included in the subsequent analysis. Other ASVs not matching the COI database, with sequence identity ≥ 0.8 and minimum query coverage ≥ 0.9 for forward and reverse reads, were filtered out by using the Basic Local Alignment Search Tool N [58]. Further, a tolerance of 30-bp distance from the 5ˈ/3ˈ end of the target sequence was defined for matches to be included. After removal of non-matching ASVs, samples with less than 500 remaining reads were removed (*n* = 16), resulting in 46 samples (*n* = 32 foal samples, *n* = 14 mare samples) from 21 individuals (*n* = 16 unique foals, *n* = 5 unique mares) that were included for further analysis. Before taxonomic classification, all spurious ASVs with a frequency below 0.1% across all samples were removed (*n* = 208). The COI database was then used for the taxonomic assignment of the strongyle species [43]. The species were assigned if both the forward and reverse read had ≥ 80% matching identity and ≥ 90% query coverage when using both rdp (minBoot = 0.8) and vsearch version 2.27 (sintax_cutoff = 0.8). A tolerance of 30-bp distance from the 5ˈ/3ˈ end of the target sequence was defined for matches to be included. The taxonomic assignments from both the rdp and vsearch tools were used to reach a consensus, with conflicting assignments between the two tools considered ‘unclassifiable’. Multiple ASVs were assigned for a single species, where necessary.

### Statistical analysis

R (versions 3.1.4 and 4.4.1) was used for all of the statistical analyses and visualisations [59]. Horses were enrolled in the study over a period of 3 weeks, depending on their date of birth and individual start of grazing. Since the goal was to investigate mixed infections over the grazing period, FEC data were analysed on an absolute time scale. For this purpose, the first day a horse was included in the study was fixed as day 0. Since multiple samples from different timepoints were available per individual, species frequency was used to describe compositional differences between groups. Differences in species frequency between foals and mares were analysed using a mid-p exact test (R exact2×2 package, version 1.6.9), after creating 2 × 2 contingency tables of positive and negative counts per group for each strongyle species. The false discovery rate method was used to correct for multiple testing (R stats package, version 4.4.1). Furthermore, Wilson’s binomial confidence intervals (CIs) were calculated for the proportional frequency of each classified species (R epitools package, version 0.5–10.1). Unclassified species were excluded from the species frequency analysis. A univariate generalized linear model with a binomial distribution (R lme4 package, function glmer, version 1.1–35.5) [60] was used to analyse all of the relevant variables for dependent variable strongyle egg presence/absence (mds_bin). These included last administered anthelmintic drug (anthelmintic); days since the last anthelmintic treatment (deworm_days); days since the start of grazing (grass_days); timepoint, 1–11 (sample); mare or foal (group); and presence/absence of ascarid eggs (pa_bin). To account for repeated measures and farm-level variability, an individual horse identifier (horse_id) and farm identifier (farm) were included as random effects, after testing model improvement with a χ^2^ test (R lme4 package, function anova, version 1.1-35-5). Conditional and marginal* R*^2^ values were determined for each variable by using the Nakagawa method, to assess the explanatory variance of the fixed effects with and without the random effect (R performance package, version 0.13.0). Beta coefficients were exponentiated to odds ratios (ORs), and 95% CIs were calculated using the Wald method. In addition, a generalized linear mixed model built using the Template Model Builder (glmmTMB), with a binomial distribution, was used to test influence factors on mds_bin (R glmmTMB package, version 1.1.10) [61]. Horse_id and farm were included as random effects. Due to collinearity, grass_days was excluded from this model (R corr package, version 0.4.4) [62]. The remaining variables were included as fixed effects. Then, backward elimination of non-significant variables through likelihood ratio tests was performed (R lme4 package, function drop1, version 1.1-37) and model fit visualisations (R DHARMa package, version 0.4.7) [63]. ORs were calculated by exponentiating beta coefficients from the glmmTMB model, and 95% CIs were calculated using the uniroots method (R glmmTMB package, version 1.1.10). Results with CIs excluding 1 and *P*-values < 0.05 were considered significant. Nakawaga’s* R*^2^ method was used to assess the variance explained by fixed and random effects in the final model (R performance package, version 0.13.0).

The total number of assigned species per host was compared between groups by using a* t*-test (R stats package, version 4.4.1), as the data showed a near normal distribution in a histogram (R vegan package, version 2.6.1.) [64]. A non-normal distribution was assumed for all other input data after visual inspection of the histograms and the Kolmogorov–Smirnov test (R vegan package, version 2.6-8).

Alpha diversity was analysed using Chao1 richness, inverse Simpson and the Shannon index (R phyloseq package, version 1.41.1). A univariate generalized linear mixed effect model (glmer), assuming a gamma distribution with a log link function (R lme4 package, version 1.1.-37), was calculated for each of the three alpha diversity measures. This included anthelmintic, deworm_days, farm, group, mds_epg (strongyle FEC) and pa_bin as fixed effect variables. Sample was included as a random effect variable to account for repeated measures. Conditional and marginal* R*^2^ values were determined for each variable by using the Nakagawa method, to assess the explanatory variance of the fixed effects with and without the random effect (R performance package, version 0.13.0). The model’s beta coefficients were exponentiated to rate ratios and 95% CIs were calculated using the profile likelihood method. In addition, a generalized multivariate linear mixed-effects model (glmmTMB) was calculated assuming a gamma distribution with a log link function (R glmmTMB package, version 1.1.10), with sample set as random effect. Due to collinearity, pa_bin was excluded from the multivariate models. Then, backward elimination of non-significant variables through likelihood ratio tests was performed for the individual models of the three alpha diversity measures (R lme4 package, version 1.1.-37). Final model fits were tested regarding uniformity and overdispersion (R DHARMa package, version 0.4.7). Beta coefficients were exponentiated to rate ratios, and 95% CIs were calculated by using the Wald method (R performance package, version 0.13.0). Furthermore, Nakagawa’s* R*^2^ was calculated to assess the variance explained by fixed and random effects (R performance package, version 0.13.0). In cases with very low random effect variance,* R*^2^ was measured using the get_variance function (R insight package, version 1.0.7).

For beta diversity, both Bray–Curtis dissimilarity (BCD) and Jaccard distance (JD) were calculated (R phyloseq package, version 1.44.0; R vegan package, version 2.6–8). Based on these, a permutational multivariate ANOVA was run with farm and horse_id as random effects and group as fixed effect. Furthermore, non-metric multidimensional scaling ordination plots were created (R vegan package, version 2.6–8; R ggplot2 package, version 3.5.1).

## Results

### Faecal egg counts

**Fig. 1:**
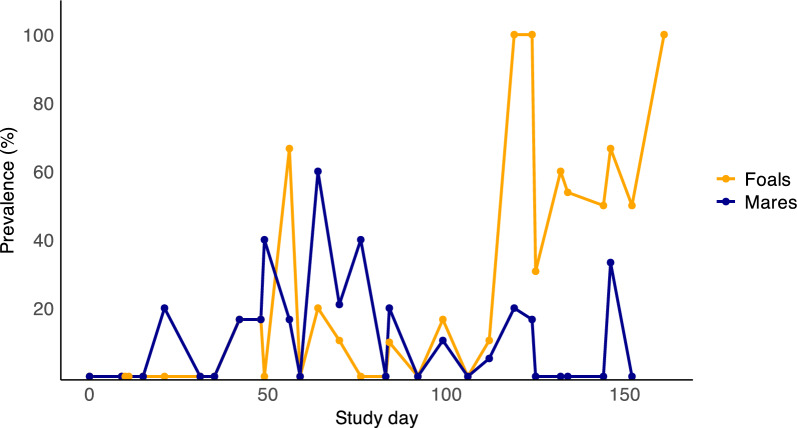
Prevalence of strongyle eggs for mares and foals over the study period, based on faecal egg counts. Samples were collected at 11 timepoints for each farm. All faecal egg counts obtained between May and November 2022 are included (*n* = 497). Samples with > 5 eggs per gram were considered positive

Coproscopic analysis of 497 samples led to the detection of three types of nematode eggs, those of strongyles (Strongylida), ascarids (*Parascaris* spp.), and *S. westeri* (*n* = 1) (Table 1). Strongyle eggs were the only nematode egg type detected in mares. Over the study period, the prevalence of strongyle eggs ranged from 0 to 100% in foals and 0–60% in mares at different timepoints throughout the study period (Fig. 1). A multivariate analysis revealed that ascarid egg detection increased the odds for strongyle egg detection by a factor of 6.49 (OR = 6.49, *P* < 0.001) (Supplemental Table 2). The odds of strongyle egg detection also increased with each sample timepoint (OR = 1.36, *P* < 0.001). Horses that were treated last with pyrantel had significantly lower odds of strongyle egg detection compared to those that had received a single-fold dose of fenbendazole (OR = 0.12, *P* = 0.002) (Supplemental Table 2).

**Table 1:** Faecal egg counts for strongyle and ascarid eggs in mares and foals, based on longitudinal faecal egg count analysis

	Strongyle eggs					Ascarid eggs				
	Positive^a^		Faecal egg count			Positive^a^		Faecal egg count		
Group	*n*	%	Median	Mean	Range	*n*	%	Median	Mean	Range
Foals (*n* = 260)	56	22.2	0	4	0–60	38	15.1	0	12	0–345
Mares (*n* = 237)	25	10.2	0	10	0–865	0	0.0	0	0	0

^a^Positive indicates eggs per gram > 5

Including both horse_id and farm as random effects significantly improved the model [Akaike information criterion (none) = 443.9, Akaike information criterion (horse_id + farm) = 421.2, *P* < 0.001]. In the multivariate model, 63.1% of the variance was explained by random effects (Nakagawa’s *R*^2^, conditional *R*^2^ = 0.634, marginal *R*^2^ = 0.498).

### Anthelmintic treatments

The most recent anthelmintic treatment at the time of sample collection was recorded. For mares, pyrantel (*n* = 117) and ivermectin (*n* = 119) were most frequently used, followed by a single-fold dose of fenbendazole (*n* = 6) and moxidectin (*n* = 3). Foals had last been treated with a fivefold dose of fenbendazole (*n* = 115), a single-fold dose of fenbendazole (*n* = 107), ivermectin (*n* = 19) or pyrantel (*n* = 11). Prior to this study, two of the farms had never conducted faecal egg tests. The third farm had performed some diagnostic tests 1 year prior to this study after two yearlings had been hospitalized for larval cyathostominosis. While stabled, all of the horses were kept on straw bedding, which was cleaned daily and replaced as needed. Turnout areas were cleaned or harrowed at least twice a year, and lime fertilization was used once or twice a year. At one farm (farm 2), horses and cattle were rotated on the pastures where they grazed.

### Species composition

**Fig. 2:**
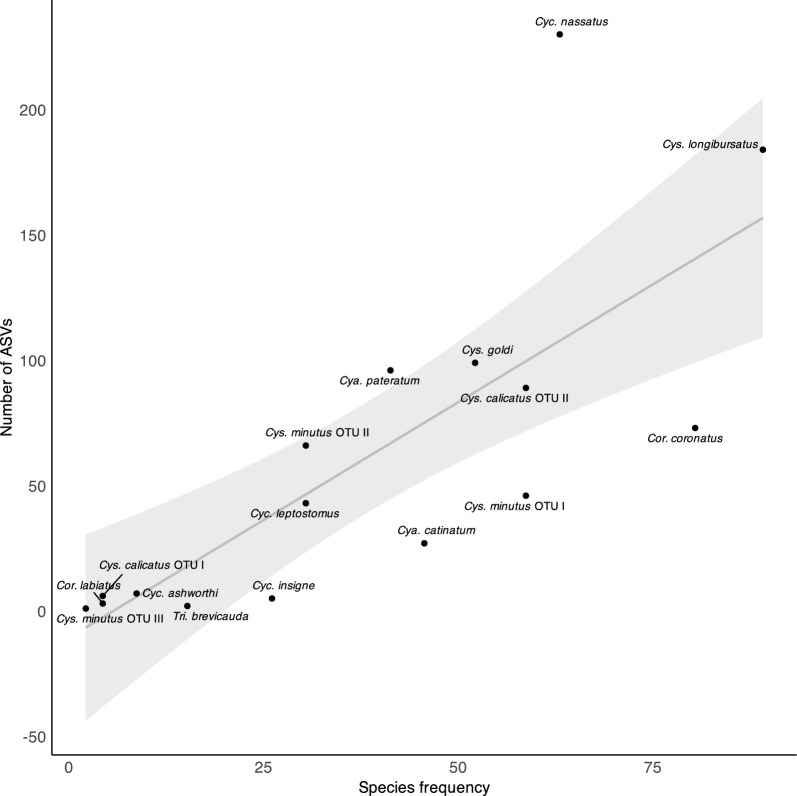
Correlation between the number of different amplicon sequence variants (ASVs) per species and overall species frequency for all of the samples (*n* = 46), based on Spearman’s rank correlation (ρ = 0.85; *P* < 0.001). The SE is shown as the grey area around the midline of the fitted trend.* OTU* Operational taxonomic unit

From a total of *N* = 1035 ASVs, *n* = 2978 were matched to 16 species of small strongyles, while 57 ASVs (5.51%) remained unclassified. Large strongyles were not detected. The highest number of ASVs was found for *Cyc. nassatus* (*n* = 230), while *Cys. minutus* OTU I had the lowest number (*n* = 1). Species frequency and number of ASVs per species were positively correlated (S = 97.75, rho = 0.85, *P* < 0.001) (Fig. 2).

Out of 16 assigned species of small strongyles, 15 were detected in foals and 13 in mares. Proportional frequencies for all species overall and for each group, as well as ORs between groups, are shown in Supplemental Table 3. In foals (*n* = 32), the median number of species of small strongyles per individual was seven (range = 2–10, mean = 6.2), whereas mares (*n* = 14) had a median of eight species of small strongyles (range = 3–9, mean = 8.0). The total number of species per host was significantly different between the two groups [*t*(23.09) = − 2.42, *P*-value = 0.023].

**Fig. 3:**
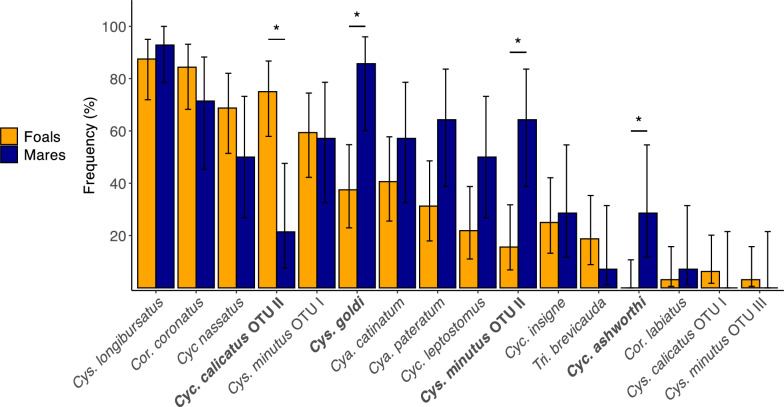
Frequency of small-strongyle species among strongyle-positive samples from mares (*n* = 14) and foals (*n* = 32). Species are sorted by descending overall prevalence (*n* = 46). Binomial confidence intervals are shown as error bars. Bold font and asterisks denote species that showed significant differences in prevalence between mares and foals, which were calculated using a mid-p exact test and the false discovery rate method to correct for multiple testing (* P < 0.05)

Four species of small strongyles showed significantly different prevalences between groups, according to the mid-p test (Fig. 3). While *Cys. goldi* was the second most prevalent species in mares (85.71%), it had a significantly lower prevalence in foals (37.5%, mid-*P* = 0.016, OR 0.11, 95% CI 0.01–0.50). Similarly, *Cys. minutus* OTU II had a significantly higher prevalence in mares (64.3%) than in foals (15.6%, mid-*P* = 0.016, OR 0.11, 95% CI 0.02–0.46). *Cylicocyclus ashworthi* was only detected in mares (28.5%, mid-*P* = 0.024, OR 0, 95% CI 0–0.42). In contrast, *Cys. calicatus* OTU II had a significantly higher prevalence in foals (75.0%, mid-*P* = 0.016, OR 10.32, 95% CI 2.40–56.43) than in mares (21.4%). Two cryptic species with a low prevalence in foals, *Cys. calicatus* OTU I (6.3%) and *Cys. minutus* OTU III (3.1%), were not detected in mares (Supplemental Table 3).

### Alpha diversity

**Fig. 4:**
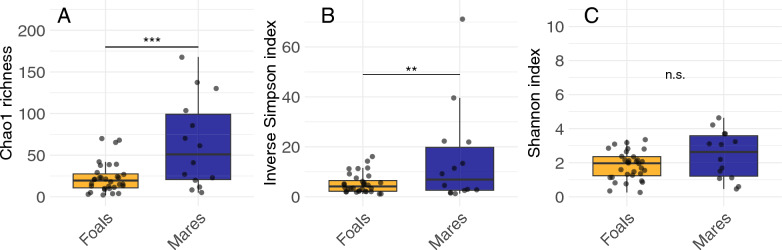
Comparison of alpha diversity of ASVs between mares (*n* = 14) and foals (*n* = 32), measuring richness through Chao1 (**A**) and diversity by inverse Simpson (**B**) and Shannon (**C**) indexes. Asterisks denote significant differences between groups, calculated using a generalized linear model with horse_id (individual identifier) and sample (timepoints 1–11) as random effect variables (**P < 0.01, ***P < 0.001,* n.s.* not significant)

Richness, measured by Chao1 and based on ASVs, was significantly higher in mares than foals (*P* < 0.001) (Fig. 4A). Diversity was significantly different between groups as well for the inverse Simpson index (*P* < 0.01) (Fig. 4B). The Shannon index was not significantly different for the two groups (*P* = 0.10) (Fig. 4C).

Random effects of horse_id (individual identifier) and sample (timepoints 1–11) were detected in both univariate and multivariate generalized linear mixed models. However, group was the only significant variable in the final multivariate model for both Chao 1 and the inverse Simpson index (Supplemental Tables 4–5). Both indexes showed higher values for mares compared to foals (Chao1, OR 2.35, 95% CI 1.23–4.48, *P* = 0.009; inverse Simpson, OR 2.49, 95% CI 1.29–4.82, *P* = 0.007). For the Shannon index, no significant effects were detected (Supplemental Table 6).

### Beta diversity

**Fig. 5:**
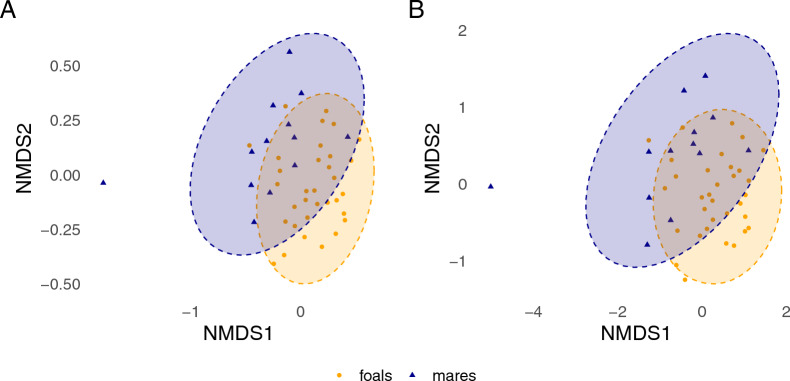
Non-metric multidimensional scaling (NMDS) ordination plots based on Bray–Curtis dissimilarity (**A**) and Jaccard distance (JD) (dissimilarity) (**B**) of ASV composition for mares (*n* = 13) and foals (*n* = 33). Points represent individual samples. Ellipses show the assumed areas in which 95% of the* t*-distribution-normalized samples of each group are located. Stress values were 0.205 for Bray–Curtis and 0.200 for JD

**Fig. 6:**
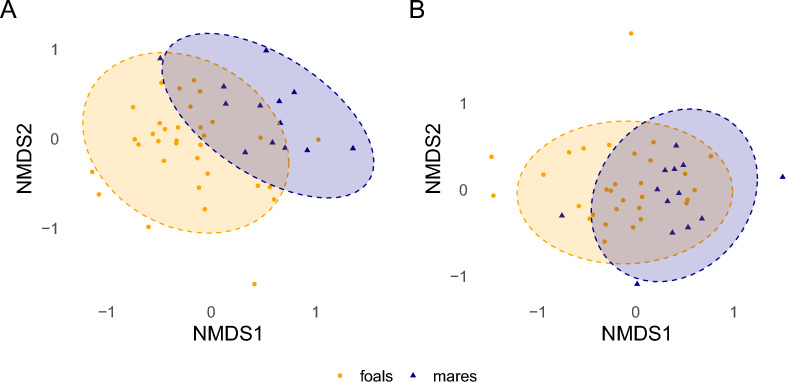
NMDS ordination plots based on Bray–Curtis (**A**) and JD (dissimilarity) (**B**) of species composition for mares (*n* = 13) and foals (*n* = 33). Points represent individual samples. Ellipses show the assumed areas in which 95% of the* t*-distribution-normalized samples of each group are located. Stress values were 0.212 for Bray–Curtis and 0.173 for JD

Based on a permutational multivariate ANOVA, both BCD and JD (dissimilarity) revealed significant differences between mares and foals (BCD, *P* < 0.01: JD, *P* < 0.01). Group explained 2.6–4.0% of the variance (BCD, *r*^2^ = 0.040; JD, *r*^2^ = 0.026). Farm was a significant random effect (*P* < 0.001), explaining 5.7–8.7% of the variance (BDC, *r*^2^ = 0.087; JD, *r*^2^ = 0.057). Sample timepoint and infection with *Parascaris* spp. showed no significant effect on beta diversity of the strongyle communities. Both BCD and JD showed almost identical results for ASV-based non-metric multidimensional scaling, with some overlap between groups and a moderate model fit (BCD, stress value = 0.200; JD, stress value = 0.205) (Fig. 5). For species-based plots, BCD (stress value = 0.141) showed more separation between clusters than JD (stress value = 0.173) (Fig. 6).

## Discussion

To the best of our knowledge, this study presents the first COI-based analysis of mixed strongyle infections in foals and broodmares. The comparison of these two groups adds to our understanding of species biology for several small strongyles, including five cryptic species. The selection of the study participants can be viewed as a unique strength of this investigation, but it also resulted in limitations that need to be considered when interpreting the results. The faecal samples were collected from horses that received anthelmintic treatment at intervals throughout the duration of the study. This type of treatment routine, which is common practice for breeding farms in Germany and elsewhere, likely contributed to the low FECs [25]. Moreover, the treatment routine may have influenced the strongyle species composition, potentially favouring resistant species and species with shorter prepatent periods. Taken together, the prevalence data from this study cannot be considered representative of the general equine population of Germany. Nevertheless, these data provide valuable insights into strongyle communities under ‘normal’ conditions in breeding stables, which is currently lacking.

Strongyle infections were less prevalent than expected in this study. This may have been related to the unusually low rainfall during the study period [65]. Lower parasite burdens have been reported in dry years [31]. Strongyle infection prevalence is generally reported to be very high worldwide in horses with access to pasture [3], but data for suckling foals are scarce. Moreover, differences between age groups [24] and anthelmintic treatment strategies [38, 66] have been described. In thoroughbred mares kept under very similar husbandry conditions to the broodmares in this study, strongyle prevalence of only 32% was reported [67]. Moreover, middle-aged and older mares (10–20 years) had significantly lower prevalences than younger mares [67].

The low FEC (< 50 EPG) found in the present study contributed to sample loss during egg isolation, as it was not possible to retrieve sufficient strongyle eggs for subsequent DNA isolation from all of the positive samples. Moreover, unusually high amounts of strongyle egg-sized faecal matter complicated egg isolation, as additional washing steps had to be included, which may have added to the loss of more eggs during their isolation. Future studies that include metabarcoding could consider using larval culture instead of egg sampling to avoid sample loss due to low egg yields. Larval culture has been used in recent studies [20, 40, 46], and was also found to be a reliable and sensitive sampling technique for characterizing strongyle infections in recent work conducted by our group [55].

Overall, foals were significantly more likely to be positive for strongyle eggs than mares, which was to be expected. Differences in strongyle infections between age groups have been well described [5, 24, 25, 28, 67], and treatment intervals and choice of anthelmintic drug may have contributed to this. During the first 4–8 weeks of life, the foals were more frequently treated (< 4-week intervals) than the mares. However, they were exclusively treated with fenbendazole during this time. Given the widespread occurrence of benzimidazole-resistance in small strongyles worldwide [12, 16, 68–71], it remains uncertain whether these treatments had any effect on the small strongyle infections that were present. However, it is possible that the differences in species composition and prevalences between the groups can be attributed to the use of different anthelmintic treatments for mares and foals. In addition, later sampling timepoints coincided with longer treatment intervals (8–12 weeks) in foals in this study, and may have contributed to the higher strongyle prevalence in them. The broodmares were almost exclusively treated with pyrantel or a macrocyclic lactone, for which varying efficacy against strongyles has been reported. Resistance to pyrantel has been described in many areas, including Germany [71]. Likewise, reports of macrocyclic lactone resistance in small strongyles are increasing in Europe [20, 72, 73] and other countries [74, 75]. However, to date, no case has been reported in Germany. In addition, differences between strongyle species in their resistance to anthelmintics have been suggested for all classes of these drugs [11, 18, 73]. Investigating species-specific anthelmintic resistance would be an interesting goal for future studies.

Horses had higher odds of testing positive for strongyle eggs with increasing time on pasture. This may have been related to the ingestion of higher volumes of roughage with the increasing age of the foals and the time factor. Both of these factors could have increased the opportunities for infection. Older foals have been shown to have higher strongyle worm burdens and egg shedding than younger foals [11, 76]. Alternatively, it may have been related to seasonality. Since all of the foals were born in the spring, it was not possible to analyse grazing time and season separately. The seasonality of strongyle egg shedding is controversial. A number of studies have reported seasonality of strongyle infections [26, 29–31], with greater differences described for young horses (< 5 years) [31]. However, a large retrospective study that used postmortem data [4], and two longitudinal studies on untreated adult equines [4, 77, 78], reported a lack of seasonality in strongyle egg shedding. Seasonality may also differ when studies are based on data from necropsies or EPGs. To the best of our knowledge, this is the first longitudinal investigation of horses receiving treatment at intervals, and hence, it remains unclear how seasonality may factor into our observations.

Sixteen non-migratory strongyle species were identified overall in this study, which is in accordance with the species richness described for other treated populations of horses in Western Europe [3]. Unsurprisingly, *Cys. longibursatus*, *Cor. coronatus* and *Cyc. nassatus* were the three most frequently detected species. They have been identified as highly prevalent and abundant species worldwide, as reported by a large multi-country meta-analysis [3]. Of note, the cryptic species *Cys. calicatus* OTU II and *Cys. minutus* OTU I were, equally, the fourth most frequently detected species overall (58.7%). This matches findings from morphological studies, which reported both morphospecies, *Cys. calicatus* and *Cys. minutus,* among the core species [3, 79]. To date, only two metabarcoding studies differentiated cryptic species in naturally occurring infections, and both found a high prevalence of *Cys. calicatus* OTU II, while *Cys. minutus* OTU I only had a low to medium prevalence, 33.3–39.4% [40, 55]. Interestingly, two other cryptic species, *Cys. minutus* OTU II and III were more prevalent than *Cys. minutus* OTU I in both of those studies, whereas they had low and very low abundance, respectively, in the present study.

While most of the identified species were detected at similar abundances in mares and foals, four species showed significant differences. First, the core species, *Cys. goldi*, was significantly less likely to be detected in foals. No age or group association has been previously described for this species. However, *Cys. goldi* was found to be resistant to pyrantel, ivermectin and moxidectin in a North American study [80]. A difference in anthelmintic response could have contributed to the dominance of this species in the mares which, unlike the foals, had been regularly treated with pyrantel and ivermectin. Although macrocyclic lactone resistance has not yet been reported in Germany, it would be interesting to examine the anthelmintic susceptibility of this species in future studies. Second, *Cyc. ashworthi* was significantly more frequently detected in mares in the present study. In fact, it did not occur in the foals at all. This contrasts with the findings of a study that reported an age association between *Cyc. ashworthi* and 2-year-old horses, and a significantly lower presence of *Cyc. ashworthi* in older horses [24]. However, the latter study did not include foals, which may explain the differences between its findings and ours. A morphological study of small strongyles in suckling foals also found a low prevalence for *Cyc. ashworthi* [11]. Third, the cryptic species *Cys. minutus* OTU II was among the most common species in the mares, and had a significantly higher frequency than in the foals. The detection frequency for this species among mares in the present study is similar to the prevalence described for treated adult horses in another study [42], and may point toward an association with age. Fourth, *Cys. calicatus* OTU II was among the most common species in foals, in which it was significantly more frequent than in mares. Interestingly, *Cys. calicatus* OTU II was also highly prevalent in a recent study on yearlings carried out in the USA by our working group [55], and may point to an association with the infected animal’s age for this genospecies. However, more studies are needed to elucidate species-specific properties of small strongyles, especially regarding the cryptic species. Taken together, these findings support the idea that these cryptic species are valid individual species within the context of the phylogenetic species concept [13, 43, 49, 81]. Moreover, they highlight the need to apply methods that can detect cryptic species, and methods and criteria that can be used to identify such strongyle species should be harmonized. Overall, our knowledge about the biology of species of small strongyles is still lacking and should be addressed in future investigations. Determining the anthelmintic susceptibility or resistance status of the identified species in future studies would be of high value, as drug efficacy likely represents a major factor influencing the occurrence of individual species. Three recent studies have investigated the species composition in strongyle populations of species with known anthelmintic resistance [73–75]. However, the anthelmintic efficacy of specific drugs against individual strongyle species is largely unknown. While it may not be feasible to test anthelmintic efficacy in strongyle species with low abundance, or that are rare, future studies could investigate the core strongyle species using a combination of a faecal egg count reduction test and molecular identification methods [33].

Strongyle community analyses revealed some interesting differences between the mares and foals. The broodmares had significantly higher ASV richness as well as a higher number of species per horse. These are interesting findings, as a previous study found higher species richness in young horses and a decline with the animals’ age [24], albeit suckling foals were not included in that study. It is possible that mixed strongyle infections develop differently in animals in the first months of life than in adults, which may explain our finding. Some studies have suggested an influence of host immunity on strongyle communities in neonates, which could potentially influence species composition and richness [11, 24]. Unlike foals, mares had likely been repeatedly infected with strongyles in the years prior to the study period. Thus, despite anthelmintic treatment, they may have harboured encysted larvae from previous infections, which could have gradually egressed and contributed to higher species richness. Body size has been found to be a strong determinator of parasite richness in many host species [82], and has been discussed with regard to the strongyle burdens of equines [24]. Alternatively, differences in richness may be related to differences in immune responses to strongyle infections. The foals, which would have been infected for the first time, would have had limited immunity towards strongyle infections, whereas the mares probably had partial immunity prior to the study through exposure in previous grazing seasons. Repeated infections promote immune maturity, which can result in resistance or tolerance towards strongyle challenge, and both responses potentially differ between individuals and with regard to strongyle species [83]. In small strongyles, several studies have shown evidence of increased host resistance with increasing age of the host, based on declining FEC magnitudes [84, 85], with the exception of young foals [78] and geriatric equines [86]. In contrast, based on the same FEC patterns, a recent study suggested that immune maturity may instead enhance tolerance to infection with small strongyles [24], allowing infections to persist without a marked clinical impact. Although this hypothesis has not been tested directly, it is consistent with observations from untreated populations, which often exhibit higher FEC and greater species richness [78, 87–89]. 

Parasite-related factors may also play a role in this, as species differ in their sensitivity to host immune responses [24], potentially contributing to variation in strongyle community composition between foals and adults. Another factor to consider is the FEC of the sequenced samples. Although broodmares had overall lower prevalences as shown by strongyle-positive samples, the samples of mares included in the deep amplicon sequencing and subsequent community analysis had significantly higher EPGs than those of the foals. This could have increased the probability of identifying rarer species, in which case, higher richness would have been a likely outcome. While strongyle EPG did not significantly contribute to species richness in the univariate model (Supplemental Table 4), a significant but very small OR increase for the inverse Simpson diversity was detected in the univariate generalized linear mixed model (Supplemental Table 5, OR = 1.01, CI = 1.00–1.01, *P* = 0.002), which potentially indicates a substantial effect due to its multiplicity with each additional egg per gram. Given that EPGs can reach several hundreds to thousands in strongyles [28, 78], the differences in EPGs between the mares and foals could have significantly impacted species diversity. This finding is in contrast to that of a previous study, which found no linear relationship between species diversity and FECs [24]. In addition to higher richness, the mares showed a significantly higher diversity of strongyles based on the inverse Simpson index, which points to differences in the dominant species between the groups. This was also seen in the direct comparison of individual species’ occurrences between the groups, and may indicate an age group association for some species. The difference in dominance structure in the present study contrasts with a reported lack of difference in species dominance, despite richness differences between age groups of infected animals [24]. However, as previously mentioned, the latter study did not include suckling foals, which limits the comparison. Moreover, our study was limited by the overall low strongyle prevalence. While the same diversity trend was observed regarding the Shannon index, differences between mares and foals were not significant here, suggesting that the less frequent species were similar in both groups. Both ASV-based and species-based beta diversity analysis showed that group explained some strongyle community differences. However, while statistically significant, only a low beta diversity variance was explained by group, suggesting that additional factors influenced strongyle community composition. Interestingly, temporal changes did not show a significant effect on beta diversity. It must be noted that a median and mean of only two samples per individual were available for this analysis, due to the overall low prevalence of strongyle eggs throughout this longitudinal study. More studies are thus needed to improve our understanding of the factors that shape strongyle communities.

## Conclusions

This first, to the best of our knowledge, COI-based characterization of mixed strongyle infections in broodmares and foals revealed differences in strongyle communities between these groups. Possible associations with age of the animals were identified for four of the species of small strongyles, including two cryptic species. However, several factors, including low overall strongyle prevalence and differences in anthelmintic treatment, limited interpretation of the results. Nevertheless, our findings contribute to an understanding of species-specific properties and strongyle community structure in broodmares and foals kept under conventional husbandry conditions. Future research is needed to improve our understanding of host-parasite interactions between horses and strongyles across age and treatment groups, and to investigate anthelmintic resistance at the species level.

## Supplementary Information

Additional file1 (PDF 48 KB)

Additional file2 (XLSX 57 KB)

Additional file3 (DOCX 48 KB)

Additional file4 (XLSX 15 KB)

## Data Availability

The dataset supporting the conclusions of this article is available under BioProject PRJNA1284338. Key programming scripts are available at 10.6084/m9.figshare.29108408.

## Abbreviations

ASV: Amplicon sequence variant
BCD: Bray–Curtis dissimilarity
CI: Confidence interval
COI: Cytochrome *c* oxidase subunit I
*Cor.** coronatus*: *Coronocyclus coronatus*
*Cor.** labiatus*: *Coronocyclus labiatus*
*Cor. ** labratus*: *Coronocyclus labratus*
*Cya.** catinatum*: *Cyathostomum catinatum*
*Cya. ** pateratum*: *Cyathostomum pateratum*
*Cyc.** ashworthi*: *Cylicocyclus ashworthi*
*Cyc.** insigne*: *Cylicocyclus insigne*
*Cyc.** leptostomum*: *Cylicocyclus leptostomum*
*Cyc.** nassatus*: *Cylicocyclus nassatus*
*Cys.** calicatus*: *Cylicostephanus calicatus*
*Cys.** goldi*: *Cylicostephanus goldi*
*Cys.** longibursatus*: *Cylicostephanus longibursatus*
*Cys.** minutus*: *Cylicostephanus minutus*
FEC: Faecal egg count
JD: Jaccard distance
OR: Odds ratio
OTU: Operational taxonomic unit
