# Supplementary figures and images for: Characterizing mixed strongyle infections in foals and broodmares using cytochrome c oxidase subunit I deep amplicon sequencing
Source: Parasit Vectors. 2026 Jan 3;19:65. doi: 10.1186/s13071-025-07192-1 (PMC12866475; doi:10.1186/s13071-025-07192-1)

Supplements Figure 1. Overview of included and excluded samples

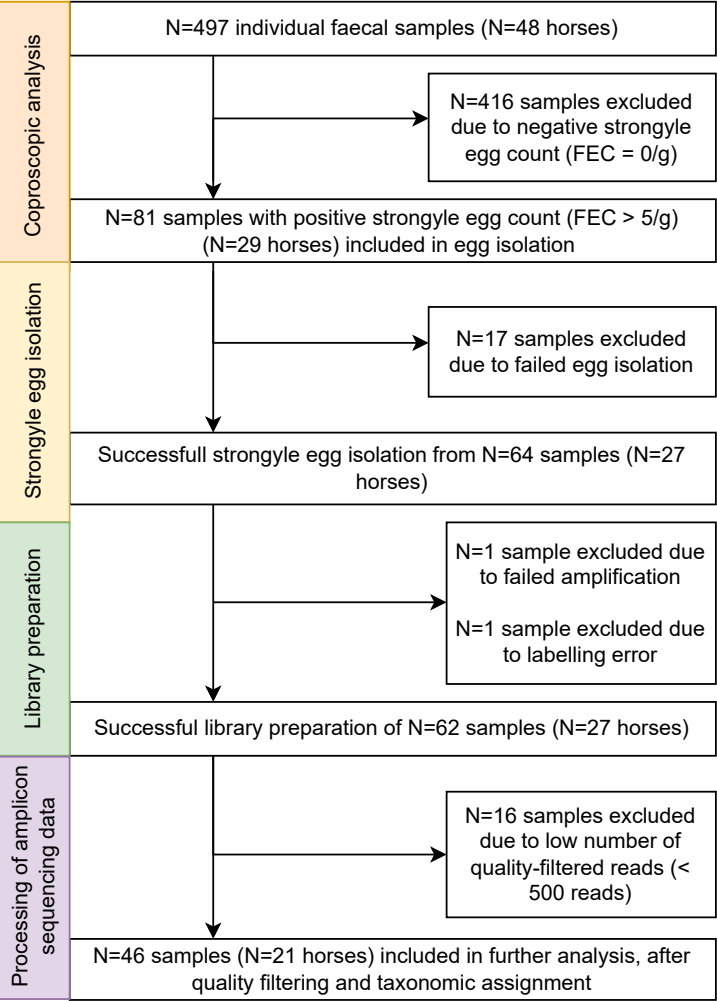

Supplement: Supplementary file 1 — Additional file1 (PDF 48 KB) [file 13071_2025_7192_MOESM1_ESM.pdf]
