# Supplementary material for: Characterizing mixed strongyle infections in foals and broodmares using cytochrome c oxidase subunit I deep amplicon sequencing
Source: Parasit Vectors. 2026 Jan 3;19:65. doi: 10.1186/s13071-025-07192-1 (PMC12866475; doi:10.1186/s13071-025-07192-1)
Supplement: Supplementary file 3 — Additional file3 (DOCX 48 KB) [file 13071_2025_7192_MOESM3_ESM.docx]

**Supplements Table 2. Factors influencing strongyle egg detection (mds_bin = 1) in horses (n=48) in univariable and multivariable generalized mixed models, based on longitudinal faecal egg count analysis (N=497).**

|  | | | **Univariable model** | | | | | | |  | **Multivariable model** | | | | |
| --- | --- | --- | --- | --- | --- | --- | --- | --- | --- | --- | --- | --- | --- | --- | --- |
| **Variable** | **Level** | **Effect** | **ß** | **OR** | **95% CI** | | **p-value** | **R^2^(m)** | **R^2^(c)** | **ß** | **OR** | **95% CI** | | **p-value** | **R^2^** |
| Anthelm* | fen1 | Fixed | 0.77 | 0.77 | 0.21 | 2.86 | 0.691 | 0.32 | 0.54 | -3.64 | 0.026 | 0.004 | 0.178 | 0.000 |  |
| Anthelm* | fen5 | Fixed | 0.12 | 0.12 | 0.04 | 0.31 | <0.001 | 0.32 | 0.54 | -0.52 | 0.596 | 0.152 | 2.289 | 0.448 |  |
| Anthelm* | ivm | Fixed | 0.20 | 0.20 | 0.08 | 0.52 | <0.001 | 0.32 | 0.54 | -0.01 | 0.990 | 0.281 | 3.689 | 0.988 |  |
| Anthelm* | mox | Fixed | <0.001 | <0.001 | <0.001 | 1.30E+130 | 0.913 | 0.32 | 0.54 | -20.20 | 0.000 |  | 0.441 | 0.998 |  |
| Anthelm* | pyr | Fixed | 0.13 | 0.13 | 0.04 | 0.37 | <0.001 | 0.32 | 0.54 | -2.14 | 0.118 | 0.027 | 0.420 | 0.002 |  |
| Deworm | Intercept | Fixed | 0.09 | 0.09 | 0.03 | 0.25 | <0.001 | 0.03 | 0.33 |  |  |  |  |  |  |
| Deworm | 12-157d | Fixed | 1.01 | 1.01 | 1.00 | 1.02 | 0.014 | 0.03 | 0.33 |  |  |  |  |  |  |
| Grass_days | Intercept | Fixed | 0.01 | 0.01 | 0.00 | 0.04 | <0.001 | 0.23 | 0.47 |  |  |  |  |  |  |
| Grass_days | 39-179d | Fixed | 1.03 | 1.03 | 1.02 | 1.04 | <0.001 | 0.23 | 0.47 |  |  |  |  |  |  |
| Group | foal | Fixed | 0.31 | 0.31 | 0.13 | 0.75 | 0.010 | 0.07 | 0.26 |  |  |  |  |  |  |
| Group | mare | Fixed | 0.32 | 0.32 | 0.16 | 0.65 | <0.001 | 0.07 | 0.26 |  |  |  |  |  |  |
| Sample | Intercept | Fixed | 0.02 | 0.02 | 0.00 | 0.06 | <0.001 | 0.18 | 0.47 | 0.31 | 1.359 | 1.187 | 1.581 | <0.001 |  |
| Sample* | 1-11 | Fixed | 1.42 | 1.42 | 1.28 | 1.58 | <0.001 | 0.18 | 0.47 |  |  |  |  |  |  |
| Pa_bin | no | Fixed | 0.13 | 0.13 | 0.06 | 0.32 | <0.001 | 0.12 | 0.31 |  |  |  |  |  |  |
| Pa_bin* | yes | Fixed | 17.56 | 17.56 | 7.53 | 40.92 | <0.001 | 0.12 | 0.31 | 1.87 | 6.495 | 2.560 | 17.221 | <0.001 |  |
| Horse_id |  | Random |  |  |  |  |  |  |  |  |  | NA | NA | 0.016 |  |
| Farm |  | Random |  |  |  |  |  |  |  |  |  | NA | NA | 0.004 |  |
| Conditional (all effects) | | |  |  |  |  |  |  |  |  |  |  |  |  | 0.631 |
| Marginal (fixed effects only) | | |  |  |  |  |  |  |  |  |  |  |  |  | 0.468 |

ß = beta coefficient; OR = odds ratio, CI = confidence interval; Anthelm = last anthelmintic drug; fen-1 = fenbendazole single-fold dose; fen-5 = fenbendazole 5-fold dose; ivm = ivermectin; mox = moxidectin; pyr = pyrantel-embonate, Deworm = days since last anthelmintic treatment; Grass_days = days since grazing start; Group = mare or foal; Sample = sampling timepoint; Pa_bin = ascarid faecal egg detection; Intercept = reference value; ^I^ = intercept for categorical variables; * = included in final multivariable model; R^2^ = variance explained, NA = not applicable.

**Supplements Table 3. Frequency of species detection overall and for groups, based on deep amplicon sequencing (N=46).**

|  | Overall (N=46) | | | Foals (n=32) | | | Mares (n=14) | | |
| --- | --- | --- | --- | --- | --- | --- | --- | --- | --- |
| **Species** | **Frequency** | **95% CI** | | **Frequency** | **95% CI** | | **Frequency** | **95% CI** | |
| *Cys. longibursatus* | 0.89 | 0.77 | 0.95 | 0.88 | 0.72 | 0.95 | 0.93 | 0.69 | 0.99 |
| *Cor. coronatus* | 0.80 | 0.67 | 0.89 | 0.84 | 0.68 | 0.93 | 0.71 | 0.45 | 0.88 |
| *Cys. calicatus* OTU II | 0.59 | 0.44 | 0.72 | 0.75 | 0.58 | 0.87 | 0.21 | 0.08 | 0.48 |
| *Cyc. nassatus* | 0.63 | 0.49 | 0.75 | 0.69 | 0.51 | 0.82 | 0.5 | 0.27 | 0.73 |
| *Cys. minutus* OTU I | 0.59 | 0.44 | 0.72 | 0.59 | 0.42 | 0.74 | 0.57 | 0.33 | 0.79 |
| *Cya. catinatum* | 0.46 | 0.32 | 0.60 | 0.41 | 0.26 | 0.58 | 0.57 | 0.33 | 0.79 |
| *Cys. goldi* | 0.52 | 0.38 | 0.66 | 0.38 | 0.23 | 0.55 | 0.86 | 0.6 | 0.96 |
| *Cya. pateratum* | 0.41 | 0.28 | 0.56 | 0.31 | 0.18 | 0.49 | 0.64 | 0.39 | 0.84 |
| *Cyc. insigne* | 0.26 | 0.16 | 0.40 | 0.25 | 0.13 | 0.42 | 0.29 | 0.12 | 0.55 |
| *Cyc. leptostomus* | 0.30 | 0.19 | 0.45 | 0.22 | 0.11 | 0.39 | 0.5 | 0.27 | 0.73 |
| *Tri. brevicauda* | 0.15 | 0.08 | 0.28 | 0.19 | 0.09 | 0.35 | 0.07 | 0.01 | 0.31 |
| *Cys. minutus* OTU II | 0.30 | 0.19 | 0.45 | 0.16 | 0.07 | 0.32 | 0.64 | 0.39 | 0.84 |
| *Cys. calicatus* OTU I | 0.04 | 0.01 | 0.15 | 0.06 | 0.02 | 0.2 | 0 | 0 | 0.22 |
| *Cor. labiatus* | 0.04 | 0.01 | 0.15 | 0.03 | 0.01 | 0.16 | 0.07 | 0.01 | 0.31 |
| *Cys. minutus* OTU III | 0.02 | 0.00 | 0.11 | 0.03 | 0.01 | 0.16 | 0 | 0 | 0.22 |
| *Cyc. ashworthi* | 0.09 | 0.03 | 0.20 | 0 | 0 | 0.11 | 0.29 | 0.12 | 0.55 |

Frequency = frequency of detection; CI = binomial confidence interval; OR = odds ratio; p = p-value; *Cor.* = *Coronocyclus*; *Cya.* = *Cyathostomum; Cyc.* = *Cylicocyclus*; *Cys.* = *Cylicostephanus*; *Tri.* = *Triodontophus*.

**Supplements Table 4. Factors influencing richness (Chao1) of strongyle communities in horses in univariable and multivariable generalized linear mixed models, based on deep amplicon sequencing of strongyle eggs (N=46).**

|  | | | **Univariable model** | | | | | | | **Multivariable model** | | | | | | |
| --- | --- | --- | --- | --- | --- | --- | --- | --- | --- | --- | --- | --- | --- | --- | --- | --- |
| **Variable** | **Level** | **Effect** | **beta** | **RR** | **95% CI** | | **P-value** | **R^2^(m)** | **R^2^(c)** | **beta** | **RR** | **95% CI** | | **P-value** | **R^2^(m)** | **R^2^(c)** |
| Anthelm | fen1 ^I^ | Fixed | 3.18 | 24.15 | 13.37 | 43.64 | <0.001 | 0.11 | 0.35 |  |  |  |  |  |  |  |
| Anthelm | fen5 | Fixed | 0.66 | 1.94 | 0.78 | 4.82 | 0.155 | 0.11 | 0.35 |  |  |  |  |  |  |  |
| Anthelm | ivm | Fixed | 0.50 | 1.65 | 0.77 | 3.53 | 0.201 | 0.11 | 0.35 |  |  |  |  |  |  |  |
| Anthelm | pyr | Fixed | -0.18 | 0.84 | 0.26 | 2.64 | 0.761 | 0.11 | 0.35 |  |  |  |  |  |  |  |
| Deworm | Intercept | Fixed | 2.95 | 19.18 | 19.02 | 19.34 | <0.001 | 0.09 | 0.39 |  |  |  |  |  |  |  |
| Deworm | 12-157d | Fixed | 0.01 | 1.01 | 1.00 | 1.01 | <0.001 | 0.09 | 0.39 |  |  |  |  |  |  |  |
| Farm | 1 ^I^ | Fixed | 3.50 | 33.26 | 18.85 | 58.67 | <0.001 | 0.02 | 0.39 |  |  |  |  |  |  |  |
| Farm | 2 | Fixed | -0.16 | 0.86 | 0.44 | 1.65 | 0.641 | 0.02 | 0.39 |  |  |  |  |  |  |  |
| Farm | 3 | Fixed | 0.17 | 1.18 | 0.63 | 2.23 | 0.601 | 0.02 | 0.39 |  |  |  |  |  |  |  |
| Group* | foal ^I^ | Fixed | 3.92 | 50.19 | 49.47 | 50.92 | <0.001 | 0.17 | 0.38 | 4.04 | 56.98 | 32.75 | 96.54 | <0.001 | 0.22 | 0.35 |
| Group* | mare | Fixed | -0.72 | 0.49 | 0.48 | 0.50 | <0.001 | 0.17 | 0.38 | -0.85 | 0.43 | 0.22 | 0.81 | 0.010 | 0.22 | 0.35 |
| Horse_id | Intercept | Fixed | 3.57 | 35.44 | 35.18 | 35.71 | <0.001 | 0.00 | 0.40 |  |  |  |  |  |  |  |
| Horse_id | 1-46 | Fixed | 0.00 | 1.00 | 1.00 | 1.00 | 0.637 | 0.00 | 0.40 |  |  |  |  |  |  |  |
| Mds_epg | Intercept | Fixed | 3.31 | 27.27 | 17.42 | 42.68 | <0.001 | 0.11 | 0.36 |  |  |  |  |  |  |  |
| Mds_epg | 5-390 | Fixed | 0.00 | 1.00 | 1.00 | 1.01 | 0.056 | 0.11 | 0.36 |  |  |  |  |  |  |  |
| Pa_bin | no ^I^ | Fixed | 3.57 | 35.51 | 35.25 | 35.76 | <0.001 | 0.02 | 0.38 |  |  |  |  |  |  |  |
| Pa_bin | yes | Fixed | -0.28 | 0.76 | 0.42 | 1.35 | 0.345 | 0.02 | 0.38 |  |  |  |  |  |  |  |
| Sample | 1-11 | Random |  |  |  |  |  |  |  |  |  |  |  |  |  |  |

ß = beta coefficient; RR = rate ratio; CI = confidence interval; Anthelm = last anthelmintic drug; fen-1 = fenbendazole single-fold dose; fen-5 = fenbendazole 5-fold dose; ivm = ivermectin; mox = moxidectin; pyr = pyrantel-embonate, Deworm = days since last anthelmintic treatment; Group = mare or foal; Sample = sampling timepoint; Pa_bin = ascarid faecal egg detection; Intercept = reference value; ^I^ = intercept for categorical variables; * = included in final multivariable model; R² = variance explained; R² (m) = variance explained by fixed effect variable (marginal R²); R² (c) = variance explained by fixed and random effect variables (conditional R²).

**Supplements Table 5. Factors influencing Inverse Simpson index of strongyle communities in horses in univariable and multivariable generalized linear mixed models, based on deep amplicon sequencing of strongyle eggs (n=46).**

|  | | | **Univariable model** | | | | | | | **Multivariable model** | | | | | | |
| --- | --- | --- | --- | --- | --- | --- | --- | --- | --- | --- | --- | --- | --- | --- | --- | --- |
| **Variable** | **Level** | **Effect** | **beta** | **RR** | **95% CI** | | **P-value** | **R^2^(m)** | **R^2^(c)** | **beta** | **RR** | **95% CI** | | **P-value** | **R^2^(m)** | **R^2^(c)** |
| Anthelm | fen1 ^I^ | Fixed | 1.77 | 5.87 | 5.84 | 5.90 | <0.001 | 0.03 | 0.45 |  |  |  |  |  |  |  |
| Anthelm | fen5 | Fixed | 0.34 | 1.40 | 1.40 | 1.41 | <0.001 | 0.03 | 0.45 |  |  |  |  |  |  |  |
| Anthelm | ivm | Fixed | 0.37 | 1.44 | 1.44 | 1.45 | <0.001 | 0.03 | 0.45 |  |  |  |  |  |  |  |
| Anthelm | pyr | Fixed | 0.12 | 1.13 | 0.36 | 3.56 | 0.834 | 0.03 | 0.45 |  |  |  |  |  |  |  |
| Deworm | Intercept | Fixed | 1.75 | 5.73 | 5.70 | 5.75 | <0.001 | 0.01 | 0.44 |  |  |  |  |  |  |  |
| Deworm | 12-157d | Fixed | 0.00 | 1.00 | 1.00 | 1.01 | 0.078 | 0.01 | 0.44 |  |  |  |  |  |  |  |
| Farm | 1 ^I^ | Fixed | 2.24 | 9.41 | 9.37 | 9.46 | <0.001 | 0.05 | 0.43 |  |  |  |  |  |  |  |
| Farm | 2 | Fixed | -0.50 | 0.61 | 0.35 | 1.04 | 0.069 | 0.05 | 0.43 |  |  |  |  |  |  |  |
| Farm | 3 | Fixed | -0.35 | 0.71 | 0.40 | 1.24 | 0.224 | 0.05 | 0.43 |  |  |  |  |  |  |  |
| Group* | foal ^I^ | Fixed | 2.41 | 11.12 | 11.06 | 11.18 | <0.001 | 0.13 | 0.43 | 2.64 | 13.94 | 6.75 | 23.97 | <0.001 | 0.26 | 0.30 |
| Group* | mare | Fixed | -0.71 | 0.49 | 0.30 | 0.79 | 0.004 | 0.13 | 0.43 | -0.94 | 0.39 | 0.21 | 0.86 | 0.072 | 0.26 | 0.30 |
| Horse_id | Intercept | Fixed | 2.12 | 8.36 | 8.32 | 8.39 | <0.001 | 0.02 | 0.44 |  |  |  |  |  |  |  |
| Horse_id | 1-46 | Fixed | 0.00 | 1.00 | 1.00 | 1.00 | 0.220 | 0.02 | 0.44 |  |  |  |  |  |  |  |
| Mds_epg | Intercept | Fixed | 1.69 | 5.42 | 3.90 | 7.52 | <0.001 | 0.27 | 0.34 |  |  |  |  |  |  |  |
| Mds_epg | 5-390 | Fixed | 0.01 | 1.01 | 1.00 | 1.01 | 0.002 | 0.27 | 0.34 |  |  |  |  |  |  |  |
| Pa_bin | no ^I^ | Fixed | 2.07 | 7.90 | 7.87 | 7.93 | <0.001 | 0.03 | 0.46 |  |  |  |  |  |  |  |
| Pa_bin | yes | Fixed | -0.36 | 0.70 | 0.38 | 1.27 | 0.242 | 0.03 | 0.46 |  |  |  |  |  |  |  |
| Sample | 1-11 | Random |  |  |  |  |  |  |  |  |  |  |  |  |  |  |

ß = beta coefficient; RR = rate ratio; CI = confidence interval; Anthelm = last anthelmintic drug; fen-1 = fenbendazole single-fold dose; fen-5 = fenbendazole 5-fold dose; ivm = ivermectin; mox = moxidectin; pyr = pyrantel-embonate, Deworm = days since last anthelmintic treatment; Group = mare or foal; Sample = sampling timepoint; Pa_bin = ascarid faecal egg detection; Intercept = reference value; ^I^ = intercept for categorical variables; * = included in final multivariable model; R² = variance explained; R² (m) = variance explained by fixed effect variable (marginal R²); R² (c) = variance explained by fixed and random effect variables (conditional R²).

**Supplements Table 6. Factors influencing Shannon index of strongyle communities in horses in univariable and multivariable generalized linear mixed models, based on deep amplicon sequencing of strongyle eggs (n=46).**

|  | | | **Univariable model** | | | | | | | **Multivariable model** | | | | | | |
| --- | --- | --- | --- | --- | --- | --- | --- | --- | --- | --- | --- | --- | --- | --- | --- | --- |
| **Variable** | **Level** | **Effect** | **ß** | **RR** | **95% CI** | | **P-value** | **R^2^(m)** | **R^2^(c)** | **ß** | **RR** | **95% CI** | | **P-value** | **R^2^(m)** | **R^2^(c)** |
| Anthelm | fen1 | Fixed | 0.62 | 1.86 | 1.31 | 2.65 | <0.001 | 0.05 | 0.14 |  |  |  |  |  |  |  |
| Anthelm | fen5 | Fixed | 0.35 | 1.42 | 0.78 | 2.58 | 0.249 | 0.05 | 0.14 |  |  |  |  |  |  |  |
| Anthelm | ivm | Fixed | 0.08 | 1.09 | 0.68 | 1.74 | 0.726 | 0.05 | 0.14 |  |  |  |  |  |  |  |
| Anthelm | pyr | Fixed | 0.11 | 1.11 | 0.49 | 2.54 | 0.800 | 0.05 | 0.14 |  |  |  |  |  |  |  |
| Deworm | Intercept | Fixed | 0.55 | 1.74 | 1.13 | 2.66 | 0.011 | 0.02 | 0.13 |  |  |  |  |  |  |  |
| Deworm | 12-157d | Fixed | 0.00 | 1.00 | 1.00 | 1.01 | 0.398 | 0.02 | 0.13 |  |  |  |  |  |  |  |
| Farm | 1 | Fixed | 0.73 | 2.07 | 1.52 | 2.83 | <0.001 | 0.01 | 0.12 |  |  |  |  |  |  |  |
| Farm | 2 | Fixed | -0.10 | 0.91 | 0.59 | 1.40 | 0.654 | 0.01 | 0.12 |  |  |  |  |  |  |  |
| Farm | 3 | Fixed | 0.02 | 1.02 | 0.68 | 1.54 | 0.917 | 0.01 | 0.12 |  |  |  |  |  |  |  |
| Group* | foal | Fixed | 0.85 | 2.35 | 1.61 | 3.41 | <0.001 | 0.05 | 0.10 | 0.90 | 2.45 | 1.85 | 3.34 | <0.001 | 0.07 |  |
| Group* | mare | Fixed | -0.23 | 0.79 | 0.50 | 1.27 | 0.337 | 0.05 | 0.10 | -0.29 | 0.75 | 0.52 | 1.05 | 0.097 | 0.07 |  |
| Horse_id | Intercept | Fixed | 0.69 | 1.99 | 1.47 | 2.68 | <0.001 | 0.00 | 0.16 |  |  |  |  |  |  |  |
| Horse_id | 1-46 | Fixed | 0.00 | 1.00 | 1.00 | 1.00 | 0.776 | 0.00 | 0.16 |  |  |  |  |  |  |  |
| Mds_epg | Intercept | Fixed | 0.64 | 1.89 | 1.50 | 2.39 | <0.001 | 0.05 | 0.12 |  |  |  |  |  |  |  |
| Mds_epg | 5-390 | Fixed | 0.00 | 1.00 | 1.00 | 1.00 | 0.260 | 0.05 | 0.12 |  |  |  |  |  |  |  |
| Pa_bin | no | Fixed | 0.75 | 2.12 | 1.66 | 2.72 | <0.001 | 0.04 | 0.14 |  |  |  |  |  |  |  |
| Pa_bin | yes | Fixed | -0.20 | 0.82 | 0.55 | 1.22 | 0.324 | 0.04 | 0.14 |  |  |  |  |  |  |  |
| Sample | 1-11 | Random |  |  |  |  |  |  |  |  |  |  |  |  |  |  |

ß = beta coefficient; RR = rate ratio; CI = confidence interval; Anthelm = last anthelmintic drug; fen-1 = fenbendazole single-fold dose; fen-5 = fenbendazole 5-fold dose; ivm = ivermectin; mox = moxidectin; pyr = pyrantel-embonate, Deworm = days since last anthelmintic treatment; Group = mare or foal; Sample = sampling timepoint; Pa_bin = ascarid faecal egg detection; Intercept = reference value; ^I^ = intercept for categorical variables; * = included in final multivariable model; R² = variance explained; R² (m) = variance explained by fixed effect variable (marginal R²); R² (c) = variance explained by fixed and random effect variables (conditional R²).
